# First person – Daniel V. Guebel

**DOI:** 10.1242/bio.058767

**Published:** 2021-05-24

## Abstract

First Person is a series of interviews with the first authors of a selection of papers published in Biology Open. Daniel V. Guebel is first author on ‘Mapping the transcriptomic changes of endothelial compartment in human hippocampus across aging and mild cognitive impairment’, published in BiO.


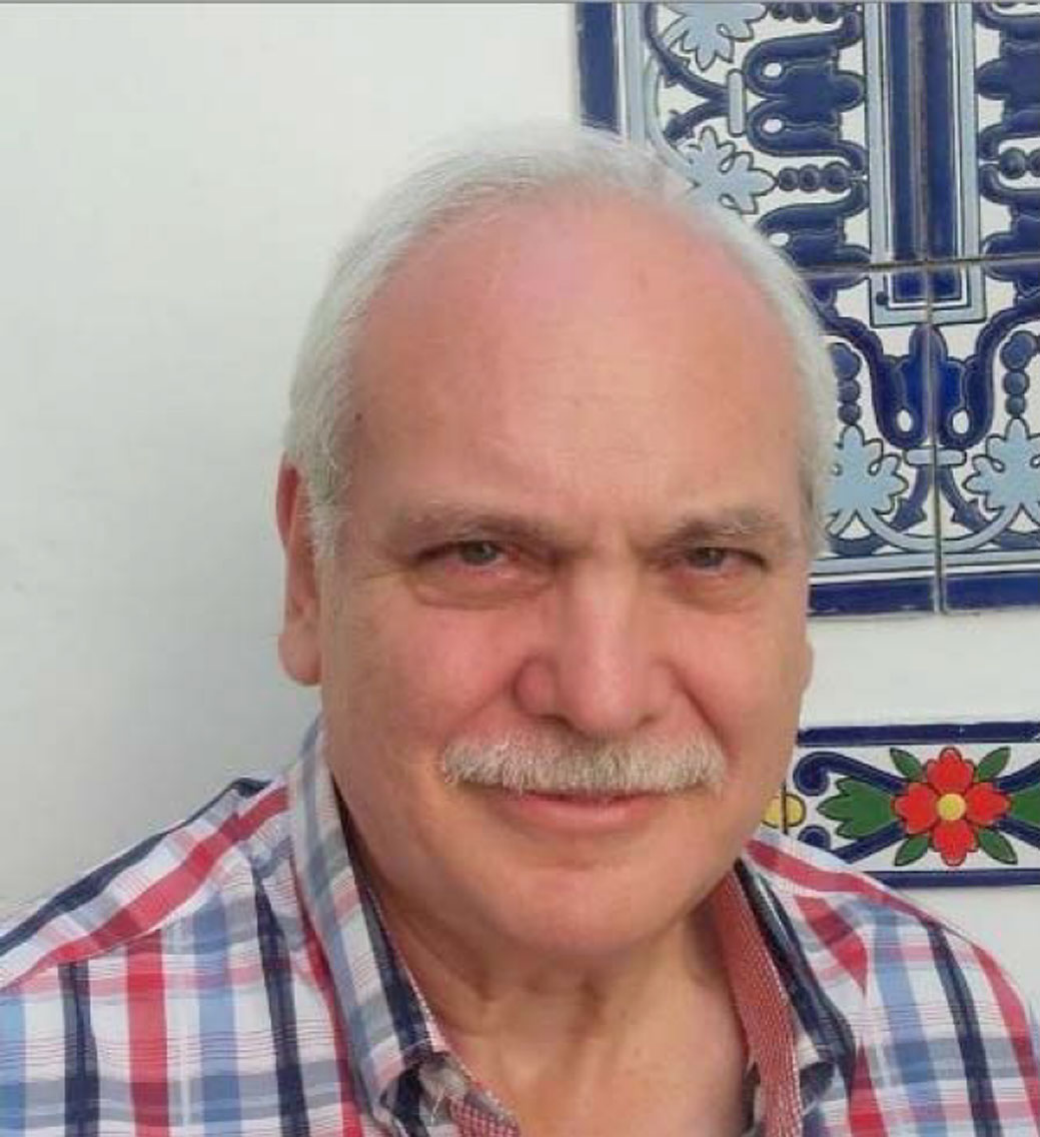


**Dr Daniel V. Guebel**

**What is your scientific background and the general focus of your lab?**

After a considerable time working in clinical chemistry as biochemist, I did a master's and a doctorate in biotechnology at the University of Buenos Aires (Argentina). From this point, I begun a career as university professor and researcher in my country. It was further continued abroad as researcher (Germany and Spain). During this time, my interest moved from the experimental work (‘wet lab’) to the (mathematical) modelling of the biological phenomena. This pursues both basic scientific objectives (i.e. a higher comprehension of the biological phenomena), as well as applied objectives (optimization for medical or industrial uses). So, my current work is in the field of the so-called ‘Systems Biology’, i.e. at the interface between physiology and the computational biology. Lastly, I was hired by the University of La Laguna (Tenerife, Spain), seeking non-invasive biomarkers for the early detection of Alzheimer's disease. There, I coordinated a multidisciplinary team to accomplish this.

**How would you explain the main findings of your paper to non-scientific family and friends?**

Hippocampus is a little, but well-structured region within the brain. It has a central role on learning and memory activities. However, hippocampal functionality can be compromised during aging, until the point to where it can be affected by neurodegenerative phenomena, as occurs in Alzheimer's disease. This disease manifests in progressive intellectual disabilities along the years, which culminate in dementia. Here, our investigation focuses on the vasculature that irrigates the human hippocampus. By computational methods we re-analysed transcriptomic data previously published concerning whole hippocampal tissue that came from three brain conditions: a) healthy, middle-aged individuals; b) healthy, elderly individuals; c) elderly individuals with mild cognitive impairment (MCI). Of note, that the data used for our analysis had not been originally designed for present use. For this reason – and to obtain inferences about how the hippocampal vasculature is functioning – we had to apply our computational approach, which performs a ‘virtual dissection’ of the whole tissue data in order to isolate the information corresponding to the vasculature. We concluded that most of the changes in the vasculature occurred previous to the apparition of cognitive disability, and even before the aging process began, i.e. in ‘neurologically healthy’ individuals. However, while in middle-age individuals, the mechanisms of compensation are still full operating (e.g. immunological surveillance, angiogenesis, response to mechanical forces, sensitivity to insulin). These functions decreased significantly during aging, conditioning the scenario for the imbalance at the MCI. Interestingly, we have observed a stage of sub-clinical inflammation (i.e. endothelial cell ‘activation’) in the healthy, middle-age individuals, coexisting with the compensatory mechanisms.

**What are the potential implications of these results for your field of research?**

Our computational approach allowed us to revise and reconcile an important number of fragmentary pieces of information, integrating them into a consistent picture that overall delineates the most plausible mode of functioning of human hippocampal vasculature under healthy and diseased conditions. As result, some new working hypotheses have been generated. Mainly, we have concluded the need to discern experimentally between the occurrence of ‘arteriogenesis’ and/or ‘capillary arteriogenesis’ as prototypic responses during normal aging, which predominate over the processes of venous specification. In any of these two instances there are a response of vascular smooth muscle cells, but vascular functionality could be compromised, since it appeared associated to increased vascular tortuosity, branching, loss of response to shear stress, increased insulin resistance, and increased response from the AGE receptors. The capillary arteriogenesis put in value the possibility of vasculature remodelling at the level of collateral circulation in the vascular anastomoses of the hippocampus, which could be an alternative mechanism to the classical model of sprouting angiogenesis. It is of note that until this time there are some transcriptional studies about the hippocampal vascularization in rodents, but not in humans.

**What has surprised you the most while conducting your research?**

A rather unexpected result that we have experienced in the present research was the finding that most important changes in the vasculature of the hippocampus do not seem to occur at the stage of MCI, but at the transition from middle-aged adulthood to elderly, being both ‘neurologically healthy’ individuals. In fact, we detected that hippocampal vasculature of middle-aged individuals is already in an ‘activated state’, i.e. in a clinical sub-inflammatory state, associated with angiogenesis. This was associated with the detection of some degree of immune response, and RAGE signals associated to HMGB1 ligand. Overall, this is contrary to the notion that in healthy individuals the hippocampal vasculature is in ‘quiescent’ stage.

**What, in your opinion, are some of the greatest achievements in your field and how has this influenced your research?**

Science building is a collective construction. In fact, in our article we have integrated and discussed more than 150 results of distinct investigations. A significant influence from this universe, but not exclusive, is the given by investigations that applied single-cell RNA-sequencing methods (sc-RNA-seq) to the vascular compartment. This methodology allows to stablish the relationship between the expression of genetic repertory in each cell type within the neurovascular unity with respect to their phenotype. However, as is discussed in the article, although certainly powerful, this technique has also some limitations. Actually, we did not apply this technique, but used a completely different approach. We believe that both approaches have to be considered as complementary.

“…in our article we have integrated and discussed more than 150 results of distinct investigations.”

**What changes do you think could improve the professional lives of early-career scientists?**

From the post-doctoral level, it would be necessary improve not only the intellectual autonomy of younger researchers, but also their financial autonomy. This could require more ample access to scientific funds, and then, a more generous attitude of welcoming from the directors of the groups, enhancing the homing of the most creative people. In the end, this would require of some significant changes in the rules and ‘traditions’ governing the scientific system.

**What's next for you?**

I will seek partners and/or support for dealing with several problems that have arisen in the field of Alzheimer's disease and other related aspects of brain aging.
